# Genetic Damage and Multi-Elemental Exposure in Populations in Proximity to Artisanal and Small-Scale Gold (ASGM) Mining Areas in North Colombia

**DOI:** 10.3390/toxics13030202

**Published:** 2025-03-11

**Authors:** Pedro Espitia-Pérez, Lyda Espitia-Pérez, Ana Peñata-Taborda, Hugo Brango, Karina Pastor-Sierra, Claudia Galeano-Páez, Gean Arteaga-Arroyo, Alicia Humanez-Alvarez, Ruber Rodríguez Díaz, Javier Salas Osorio, Luís Armando Valderrama, Tatiana Dillenburg Saint’Pierre

**Affiliations:** 1 Grupo de Investigación Biomédica y Biología Molecular, Facultad de Ciencias de la Salud, Universidad del Sinú, Montería 230001, Colombia; lydaespitia@unisinu.edu.co (L.E.-P.); investigador01gibm@unisinu.edu.co (A.P.-T.); karinapastor@unisinu.edu.co (K.P.-S.); claudiagaleano@unisinu.edu.co (C.G.-P.); geanarteaga@unisinu.edu.co (G.A.-A.); aliciahumanez@unisinu.edu.co (A.H.-A.); 2 Facultad de Educación y Ciencias, Departamento de Matemáticas, Universidad de Sucre, Sincelejo 700003, Colombia; hugo.brango@unisucre.edu.co; 3 Hospital Alma Máter, Unidad de Cuidados Intensivos (UCI), Medellín 050001, Colombia; dr.ruberrodriguez@gmail.com; 4 Hospital Alma Máter, Servicios Ambulatorios, Coordinación Médica, Medellín 050001, Colombia; salasjavier512@gmail.com; 5 Hospital César Uribe Piedrahita, Servicio de Urgencias, Caucasia 052410, Colombia; armando-090@hotmail.com; 6 Departamento de Química, Pontifícia Universidade Católica Do Rio de Janeiro (PUC-Rio), Rio de Janeiro 22453-900, Brazil; tatispierre@puc-rio.br

**Keywords:** artisanal and small-scale gold mining (ASGM), cytokinesis-block micronucleus cytome assay (CBMN-Cyt), La Mojana, GAMLSS, gold mining, multi-elemental exposure

## Abstract

This study evaluates DNA damage and multi-element exposure in populations from La Mojana, a region of North Colombia heavily impacted by artisanal and small-scale gold mining (ASGM). DNA damage markers from the cytokinesis-block micronucleus cytome (CBMN-Cyt) assay, including micronucleated binucleated cells (MNBN), nuclear buds (NBUDs) and nucleoplasmic bridges (NPB), were assessed in 71 exposed individuals and 37 unexposed participants. Exposed individuals had significantly higher MNBN frequencies (PR = 1.26, 95% CI: 1.02–1.57, *p* = 0.039). Principal Component Analysis (PCA) identified the “Soil-Derived Mining-Associated Elements” (PC1), including V, Fe, Al, Co, Ba, Se and Mn, as being strongly associated with high MNBN frequencies in the exposed population (PR = 10.45, 95% CI: 9.75–12.18, *p* < 0.001). GAMLSS modeling revealed non-linear effects of PC1, with greater increases in MNBN at higher concentrations, especially in exposed individuals. These results highlight the dual role of essential and toxic elements, with low concentrations being potentially protective but higher concentrations increasing genotoxicity. Women consistently exhibited higher MNBN frequencies than men, suggesting sex-specific susceptibilities. This study highlights the compounded risks of chronic metal exposure in mining-impacted regions and underscores the urgent need for targeted interventions to mitigate genotoxic risks in vulnerable populations.

## 1. Introduction

In recent years, Colombia has experienced a significant increase in gold production, becoming one of the top fifteen gold producers globally and one of the largest in Latin America [1]. This growth has been driven by the expansion of intensive placer mining, which involves the exploitation of open-pit and alluvial deposits [2,3]. Most of the placer mining in Colombia is carried out through a combination of artisanal and small-scale gold mining (ASGM) practices [2,4]. ASGM can be carried out formally or informally, leading to significant changes in the mining areas. Informal ASGM involves the continuous relocation of gold exploitation sites, making it a highly mobile but uncontrolled activity [5]. Additionally, ASGM results in the intensive recruitment of women, men and children who are drawn to the additional income and informal nature of this gold extraction practice [5]. These factors contribute to the success of the intensive ASGM model in Colombia.

The rise in gold prices has led to increased demand for the exportation of this mineral from Colombia. Consequently, the Colombian government has promoted the formalization of gold mining titles, leading to an increase in ASGM operations [3,6]. Unfortunately, criminal organizations also use gold profits to finance illegal activities. Since 2017, Colombia’s gold exports have greatly increased, mainly due to illegal mining [6,7]. Many unregistered mines, unrecognized by the Ministry of Mines and Energy, are employing intensive mining models that involve the use of heavy machinery, like dredgers and backhoe loaders, in most Colombian ASGM areas. This leads to the extraction of large amounts of mineral material and increases environmental risks [8]. The rise of illegal mining activities further exacerbates these environmental issues and adds to the social complexities surrounding gold production in Colombia. The effects of these operations on populations and ecosystems are unknown. Open-pit ASGM results in more human health damage due to tailings and excavation processes, while alluvial ASGM mining has significant effects on ecosystem quality from soil and vegetation stripping [3].

Intensive exploitation of mineral material in bench sides and river shores, together with mercury (Hg) amalgamation, allows the release of large amounts of toxins to the environment, affecting nearby populations. Moreover, ASGM sites are commonly associated with multi-elemental contamination in addition to Hg, which includes mainly arsenic (As), cobalt (Co), lead (Pb), manganese (Mn) and zinc (Zn) [9,10,11,12,13,14]. Inorganic As compounds are classified by the International Agency for Research on Cancer (IARC) as Category 1 carcinogens [15]. Recent studies have shown a relationship between high As concentrations in soils near ASGM activities and increased cancer risk [16,17]. Co and Pb are both Category 2B carcinogens [18,19], where Co can be related to cardiomyopathy, vision or hearing impairment, hypothyroidism, polycythemia and potential risk of lung cancer [20,21]. Pb exposure results in anemia, hypertension, disrupted brain and renal function and IQ decrements [22]. Finally, Mn overexposure leads to Parkinson-like symptoms in humans [23] and chronic Zn ingestion leads to lethargy, respiratory disorders, metal fume fever, gastrointestinal disturbances, elevated risk of prostate cancer and pancreatitis [24,25].

The northern Colombian region of “La Mojana”, which comprises the Departments of Córdoba, Sucre and Bolívar, is an area historically affected by massive ASGM activities. Data on Hg contamination has been well documented in human populations [26], fish [27,28] and plants [29]. Few studies have demonstrated the potential and biologic effects of cadmium (Cd), Pb and Hg exposure, especially in La Mojana [30,31,32]. However, no other studies have evaluated the true biological extent of ASGM in La Mojana, especially with other elemental contaminants in exposed populations, which remains elusive.

Regarding biological effects, environmental and occupational exposure to carcinogens in less-developed countries leads to an increase in cancer incidence and mortality [33], highlighting the urgent need for studies that assess cancer risk in these populations. Reflecting the advances in methods for monitoring DNA and chromosomal damage in humans exposed to genotoxicants, more recent approaches have shown a greater reliance on the evaluation of micronuclei in binucleated cells (MNBNs) and primary DNA damage [34]. The cytokinesis-block micronucleus assay (CBMN-Cyt) have been extensively used as a highly reproducible and reliable biomarker of DNA damage [35,36]. MNBNs occur when parts of chromosomes break, or entire chromosomes fail to segregate properly during cell division [37]. This process can contribute to cancer development, making MNBNs a validated marker for assessing cancer risk [33,38,39,40]. Additional biomarkers such as nucleoplasmic bridges (NPBs) are structures that form when DNA is improperly repaired or when the ends of chromosomes (telomeres) fuse; nuclear buds (NBUDs) emerge when cells attempt to remove extra or damaged DNA, indicating cellular responses to genetic stress [34,41].

Considering previous reports on increased MNBN frequencies in populations exposed indirectly to ASGM activities [42,43], this study aimed to (1) evaluate genetic damage in isolated lymphocytes of individuals living in proximity to water bodies influenced by ASGM activities from the “La Mojana” region in Colombia through the use of a CBMN-Cyt assay; (2) to assess multi-elemental exposure in exposed populations in comparison with the reference area; and (3) to determine the relationship (linear or non-linear) between genetic damage and elemental contamination in directly impacted areas.

## 2. Materials and Methods

### 2.1. Study Area

The study was conducted in several locations within the La Mojana region, a vast area comprising three departments in northern Colombia: Bolívar, Córdoba and Sucre. La Mojana is primarily populated by riparian communities, with settlements in marshlands, along small rivers and in areas that experience significant fluvial connectivity during the rainy season [42]. Previous research has shown that La Mojana has been historically affected by the extensive gold mining activities developed in southern Bolívar and northeastern Antioquia. These activities, primarily ASGM, have led to widespread contamination of aquatic ecosystems and human populations due to the release of Hg and heavy metals [44,45,46,47].

Based on previous research and field experience, we established the study areas near water bodies directly or indirectly influenced by ASGM operations or near active mining areas [42]. These sites have been previously reported to exhibit Hg and heavy metal contamination in environmental matrices, such as soil [44], water [48] and fish tissue [49]. Additionally, these areas are characterized by subsistence-based artisanal fishing among local communities. The sampling areas were established in three municipalities within La Mojana: Ayapel (Córdoba), Caimito (Sucre) and Magangué (Bolívar).

Finally, a reference site was established in the municipality of Cotorra in the Ciénaga Grande de Lorica wetland complex. This area experiences fluctuating water levels, being partially isolated during the dry season and extensive flooding during the wet season. Unlike the study sites affected by mining, the marshes and wetlands of the reference area show no documented influence from ASGM activities [50].

**Figure 1:**
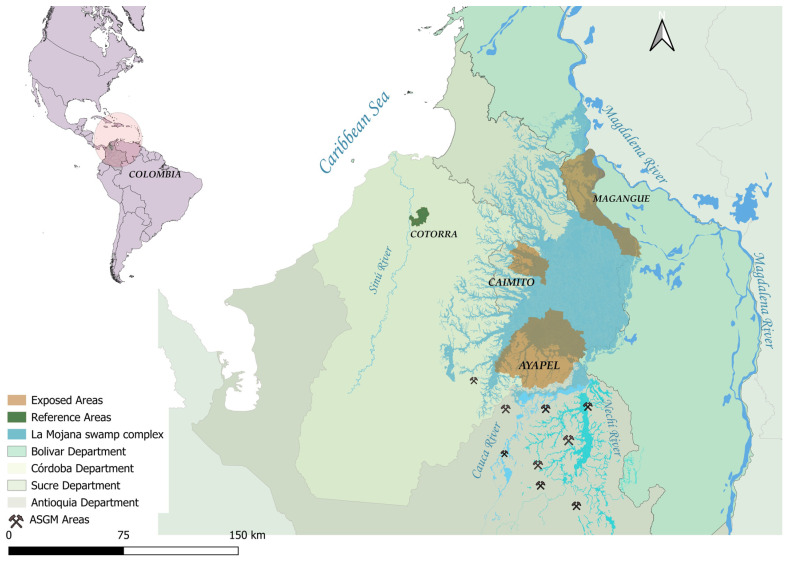
Study area in the La Mojana swamp complex in Colombia, highlighting the municipalities of Ayapel (Córdoba), Magangué (Sucre) and Caimito (Sucre), along with the main tributaries of the swamp complex. The circled area provides a centered and zoomed-in view of the study location within northern Colombia.

The exposed and reference area are situated in different river basins. The exposed sites are located within the Momposina depression (also known as the Mompox Depression), while the reference site belongs to the Sinú River Basin (Figure 1).

### 2.2. Study Population

The Research Ethics Committee of the Universidad del Sinú approved and supervised the research protocol (approval number 002/2020). A meeting was held with community representatives and municipal health authorities to present the study proposal. Participants were instructed to complete a detailed standard questionnaire that collected information on health status (including use of prescription drugs), history of cancer, other chronic diseases, lifestyle, diet, smoking habits, exposure to various pollutants, frequency of alcohol consumption, fish consumption and previous exposure to X-rays. Both exposed and reference subjects were selected on the basis of several inclusion criteria: they had to be healthy volunteers between the ages of 18 and 65 years, with no recent X-ray exposure, not using therapeutic drugs known to be mutagenic and living in the study area for at least one year. Exclusion criteria included exposure to other toxic agents, a history of genetic or respiratory diseases, the presence of dental fillings or amalgams and a family history of cancer. The study population included fishermen and their families, categorized according to the primary activity identified in the standardized questionnaire as their main source of income.

The total sample comprised 108 healthy subjects: 71 from various exposed areas and 37 from a reference area considered unexposed. Alcohol consumption was classified as light, moderate or heavy according to the guidelines of the National Institute on Alcohol Abuse and Alcoholism (NIAAA). Light drinking is defined as no more than three drinks per day and no more than seven drinks per week for women and no more than four drinks per day and no more than 14 drinks per week for men. Moderate drinking is defined as up to one drink per day for women and up to two drinks per day for men. Heavy drinking is defined as five or more drinks on the same occasion on five or more days within the past 30 days. Exposed and unexposed individuals were matched for age (±5 years), sex, socioeconomic status, occupation and ethnicity to ensure demographic comparability between groups.

### 2.3. Blood Sample Collection and Processing

Written informed consent was obtained from each participant prior to sample collection. Peripheral blood samples totaling 5 mL per individual were collected from 108 participants by venipuncture into heparinized tubes (Becton Dickinson, Franklin Lakes, NJ, USA, Vacutainer) for the CBMN-Cyt assay. All tubes were uniquely coded, stored at 4 °C, and shipped to the laboratory for testing within 24 h of collection. Blood samples from the exposed and reference groups were collected and transported simultaneously to eliminate potential variability due to different collection conditions. In addition, whole blood samples from research personnel were collected, transported, and processed under identical conditions. These were used as internal controls to identify potential confounding factors from sample handling or transport.

### 2.4. Hair Sample Collection, Preparation and Processing

Hair samples were collected from volunteer participants according to protocols described in a previous study [43]. Whole hair samples, approximately 3 cm in length and weighing approximately 500 mg, were removed from the occipital region of the scalp using stainless steel scissors. The samples were placed in 50 mL Falcon tubes labeled with a unique participant identifier. The prepared samples were treated with 2.5 mL of sub-boiled distilled nitric acid (HNO_3_) and allowed to digest overnight at room temperature. This procedure was followed by an additional digestion step at 70 °C for one hour. Hair samples were washed in an ultrasonic bath alternating between deionized water and acetone, with each solvent used for 10 min to remove exogenous contaminants. After cleaning, the samples were oven-dried at 60 °C overnight and weighed, with approximately 250 mg retained for further analysis.

### 2.5. Determination of DNA Damage and Chromosomal Instability Parameters

A mixture of 0.5 mL heparinized blood and 4.5 mL RPMI 1640 medium (Sigma, St. Louis, MO, USA) supplemented with 2 mM L-glutamine (Sigma, St. Louis, MO, USA), 10% fetal bovine serum (Gibco, São Paulo, Brazil), 100 μg/mL antibiotic-antimycotic (Sigma, St. Louis, MO, USA) and 2% phytohemagglutinin (Sigma, St. Louis, MO, USA) was prepared. Aliquots were incubated at 37 °C for 44 h under 5% CO_2_. After incubation, 6 μg/mL cytochalasin B (Sigma) was added to inhibit cytokinesis. Lymphocytes were then collected by centrifugation at 1200 rpm for 8 min, fixed in methanol/acetic acid solution (25:1, *v*/*v*), mounted on clean slides and stained with Diff-Quik (Medion Diagnostics, Düdingen, Switzerland). To assess DNA damage and chromosome instability indices, we examined 2000 binucleated (BN) cells per blood sample, with 1000 cells evaluated from each duplicate culture slide. Endpoints included micronuclei (MNBN), nucleoplasmic bridges (NPB), and nuclear buds (NBUDs), which were analyzed by light microscopy at 200–1000× magnification [51]. All slides were coded with unique identifiers to ensure unbiased analysis according to the criteria described by Fenech et al. [52].

### 2.6. Determination of Essential Trace and Toxic Elements in Hair Samples

Concentrations of essential trace elements and toxic metals in hair samples were determined by inductively coupled plasma mass spectrometry (ICP-MS) using a NexION 300X spectrometer (PerkinElmer, Sciex, Norwalk, CT, USA) as previously described [43]. The essential trace elements were copper (Cu), Mn, Fe, V, Sr, Co, Se and Zn, while the toxic metals quantified included Al, Pb, Hg, As, nickel (Ni), Cd and barium (Ba). All reagents were of analytical grade, and rhodium (200 mg·L^−1^) was used as an internal standard. The multi-element solution was purchased from Merck (Darmstadt, Germany). Each point of the calibration curve, including blank, reagent and sample, was analyzed with rhodium as the internal standard to ensure accuracy. The limits of detection (LOD) for the elements analyzed were as follows: Cu (0.019 µg·L^−1^), Mn (0.02 µg·L^−1^), Fe (4.5 µg·L^−1^), V (0.004 µg·L^−1^), Sr (0.006 µg·L^−1^), Co (0.02 µg·L^−1^), Se (0.03 mg·L^−1^), Zn (0.18 µg·L^−1^), Al (0.01 µg·L^−1^), Pb (0.001 µg·L^−1^), Hg (0.008 µg·L^−1^), As (0.004 µg·L^−1^), Ni (0.02 µg·L^−1^), Cd (0.004 µg·L^−1^) and Ba (0.04 µg·L^−1^). For quality and control, the analysis included an internal reference sample and a certified reference material (CRM). The CRM used was NCS DC73347a (human hair) analyzed by the China National Center for Iron and Steel Testing.

### 2.7. Statistical Analysis

Data were organized in Microsoft Excel, and a comprehensive quality control process was applied to address errors, missing values and outliers. This included descriptive analysis, data cleaning and management of missing data to ensure the reliability and integrity of the dataset. Categorical variables were expressed as percentages, while continuous variables, given their non-normal distributions, were summarized using medians and interquartile ranges. Quartiles were used for skewed metal concentration data. Comparative analyses between exposed and reference areas employed the Mann–Whitney U test, ensuring robust and reproducible results, with statistical significance set at *p* < 0.05. To investigate the relationships between metal concentrations and biomarkers (MNBNs, NPBs and NBUDs), a comprehensive statistical framework was applied. Initially, individual associations were assessed using Spearman’s correlation, a non-parametric method robust to non-normal distributions and effective for identifying monotonic relationships. To model the combined effects of metals, Principal Component Analysis (PCA) was employed to reduce the dimensionality of the metal concentration variables. PCA grouped the metals into principal components, capturing most of the variability in the data while addressing multicollinearity. The first four principal components (PC1-PC4) were selected based on the criterion of cumulative explained variance exceeding 60% and were subsequently used as predictors in the statistical models.

Generalized additive models for location, scale and shape (GAMLSSs) were then fitted for each biomarker as the dependent variable [53]. Before selecting GAMLSS as the primary modeling approach, we compared it with Generalized Linear Models (GLMs) using the Poisson distribution for the response variables. GLMs are a widely used statistical framework that assumes linear relationships between predictors and the response variable and models the mean response using a link function (in this case, the log link for count data). To compare the two approaches, we used the Akaike Information Criterion (AIC), a widely used metric in statistical model selection that assesses goodness of fit while balancing model fit and complexity. GAMLSS consistently showed lower AIC values across all biomarkers and subgroups (total, reference and exposed populations), indicating a better balance between model fit and parsimony compared to GLMs. GAMLSS was ultimately chosen for its flexibility in modeling non-linear relationships between the predictors and biomarkers and its ability to handle potential overdispersion and non-standard response distributions, in addition to its better fit [54,55,56]. Predictors included the principal components, exposure group (exposed or unexposed), sex and age. Penalized splines were employed to model the non-linear effects of the principal components on the biomarkers, providing a more nuanced representation of their relationships.

Prevalence ratios (PRs) with 95% confidence intervals (C95%) were derived from the GAMLSS models to quantify the relative impact of each predictor on the biomarkers [53]. The PRs were interpreted as proportional changes in the biomarkers per unit increase in the predictors, providing a detailed assessment of the combined effects of metals and covariates. To enhance interpretability, partial effect plots were generated to visualize the non-linear relationships estimated by the models [54,55,56]. This visualization facilitated a deeper understanding of the interactions between the predictors and the biomarkers.

All analyses were performed using R software (version 4.4.2 for Windows), employing the gamlss, FactoMineR and ggplot2 packages. This methodological framework ensured a robust and accurate evaluation of the complex interactions between metal mixtures and biomarkers.

## 3. Results and Discussion

### 3.1. Sociodemographic Characteristics of the Study Population

The sociodemographic characteristics and consumption habits of the population studied are detailed in Table 1. The study included 108 individuals, with 37 participants from the reference area (Cotorra, Córdoba) and 71 participants from the exposed areas (Magangué, Bolívar; Ayapel, Córdoba; and Caimito, Sucre). The population was balanced in terms of gender distribution, with women representing 54.62% of the participants (54.05% in the reference group and 54.92% in the exposed group) and men representing 45.37% (45.94% in the reference group and 45.07% in the exposed group). The mean age of the participants was 35.84 ± 10.71 years, with no significant difference between the reference group (36.51 ± 9.71) and the exposed group (35.49 ± 11.25). In the exposed areas, fishing was the primary occupation (45.07% of the individuals). Only a small subgroup of three participants reported occasional involvement in ASGM, an activity characterized by its intermittent and mobile nature known as “barequeo” (manual gold mining). Gold extraction in these cases was seasonal and involved using rudimentary methods to recover small quantities of gold, which were then sold in informal local markets. Despite their sporadic participation in ASGM, fishing remained their primary source of income and livelihood. For clarity in occupational classification, these individuals were included in the fishing category for analysis, reflecting their main economic activity while acknowledging the additional, occasional exposure to mining-related contaminants.

Consumption habits revealed generally low levels of alcohol and tobacco use. Non-drinkers represented 94.74% of the total population, with slightly higher abstinence in the reference group (91.89%) than in the exposed group (87.32%). Among the alcohol consumers, 9.25% reported low consumption, while 1.85% reported moderate consumption. Similarly, 92.59% of the participants were non-smokers, while only 7.40% were smokers (5.40% in the reference group and 8.45% in the exposed group).

Dietary habits revealed a notable dependence on fish consumption in both groups. A total of 39.81% of the participants reported frequent fish consumption (5–7 days per week), while 42.59% reported moderate fish consumption (3–4 days per week). Low fish consumption (1–2 days per week) was reported by 17.59% of the participants. In addition, 81.48% of the population included green vegetables in their diet, but only 18.51% reported regular fruit consumption, highlighting a limited intake of fruit.

### 3.2. DNA Damage and Chromosomal Instability Parameters in Exposed and Reference Populations

Analysis of DNA damage and chromosomal instability parameters in the total population (Table 2) revealed a significant increase in MNBN frequency in individuals from exposed areas (5.54 ± 3.98) compared to residents of the reference area (2.52 ± 2.12). Similarly, NPB frequencies were also significantly elevated in the exposed group (1.59 ± 3.96) relative to the reference group (0.54 ± 0.87, *p* ≤ 0.05), suggesting disturbances in chromosomal integrity and repair pathways. In contrast, NBUD frequencies remained low in both groups, with no statistically significant differences observed.

When the results were analyzed by sex, important differences emerged. Women in both the exposed and reference groups had consistently higher MNBN frequencies than men, a trend previously reported in genotoxicity studies. In the exposed group, women had a mean MNBN of 6.59 ± 4.63, while men had significantly lower values (4.28 ± 2.56, *p* ≤ 0.01). This sex-based trend was also evident in the reference group, with women showing 5.19 ± 5.04 and men showing 2.05 ± 2.63. For NPB frequencies, the difference between the exposed and reference groups was particularly evident in women. Women in the exposed group showed a significant increase (2.6 ± 5.45) compared to the reference group (0.53 ± 0.94, *p* ≤ 0.01). In men, NPB frequencies remained low and comparable between the exposed (0.58 ± 1.73) and reference groups (0.56 ± 0.80). This might suggest that women have a sex-specific susceptibility to chromosomal instability when exposed to ASGM and is consistent with previously reported evidence indicating that women typically have higher baseline levels of chromosomal damage and misrepair. This may be attributed to differences in hormonal regulation [57], DNA repair efficiency and cellular proliferation rates [58]. In contrast, NBUD frequencies did not differ significantly between groups or sexes. However, exposed men displayed slightly higher NBUD values (0.68 ± 1.28) compared to women (0.46 ± 1.02), though this difference was not statistically significant.

As shown in Table 3, MNBN, NBUD and NPB frequencies were not significantly influenced by age.

### 3.3. Concentration of Essential and Toxic Elements in Hair Samples

**Figure 2:**
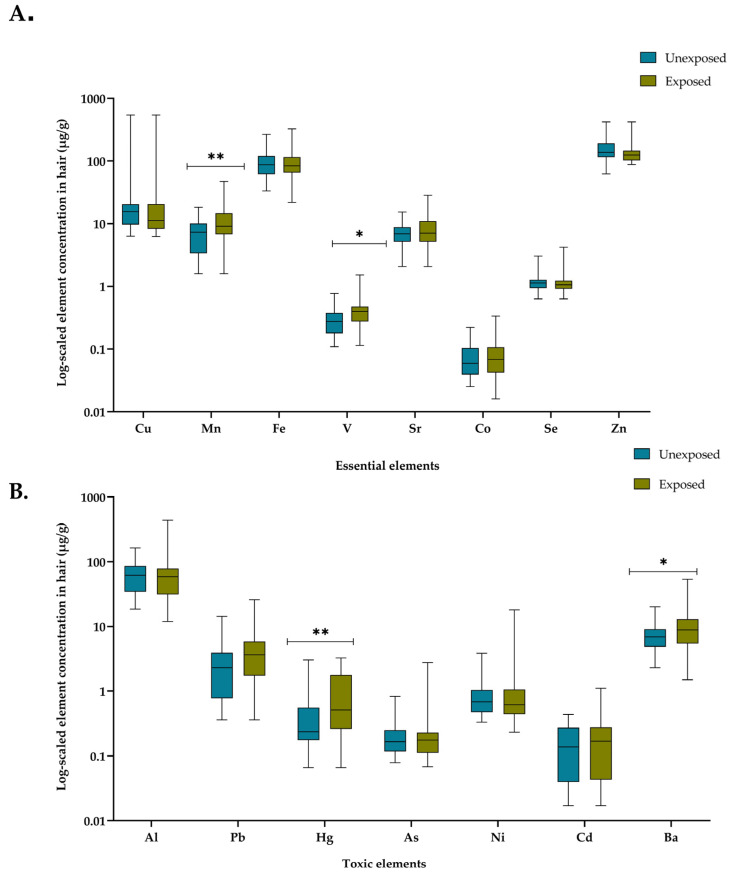
Comparison of log-scaled essential and toxic element concentrations in hair samples from exposed and unexposed populations. (**A**) Essential elements and (**B**) toxic elements are shown as boxplots, where boxes represent interquartile range (IQR), horizontal lines indicate median values and whiskers represent minimum and maximum observed concentrations. Comparisons between exposed and unexposed groups are illustrated, with statistical significance denoted as follows: *p* < 0.05 (*) and *p* < 0.01 (**).

A multi-elemental exposure assessment was performed using ICP-MS analysis on hair samples collected from individuals living in areas with no exposure and those affected by artisanal and small-scale gold mining activities. This study aimed to evaluate the extent of chronic environmental exposure by quantifying the accumulation of essential and toxic elements in both populations. The results revealed significant differences in the log-scaled element concentrations between the two groups (Figure 2).

In examining essential elements (Figure 2A), significant increases were observed in Mn and V concentrations in the exposed group when compared to the unexposed group (*p* = 0.0074 and *p* = 0.0163, respectively). While other essential elements, such as Fe and Sr, exhibited slightly elevated median levels in the exposed group, the observed differences were not statistically significant.

For toxic elements, substantial differences were observed (Figure 2B). It is noteworthy that Hg exhibited a significant increase in the exposed population (*p* = 0.0024), indicating relevant exposure likely related to local environmental factors, such as artisanal gold mining. Similarly, significant elevations were observed for Ba (*p* = 0.0428) in the exposed group. Other toxic elements, including Pb and Al, showed moderate increases but did not reach statistical significance. These findings demonstrate a clear trend of elevated concentrations of both essential and toxic elements in individuals from exposed areas, suggesting chronic environmental exposure likely linked to artisanal gold mining activities.

Building on these findings, gender-specific differences in the accumulation of essential and toxic elements were further explored. Significant variations were identified among individuals residing in the exposed areas. For essential elements, Cu concentrations were significantly higher in men compared to women (45.61 ± 127.8 mg/Kg vs. 23.55 ± 25.93 mg/Kg; *p* = 0.000251; summarized in Table S1). Conversely, Fe levels were significantly elevated in women relative to men (109.8 ± 51.15 mg/Kg vs. 80.02 ± 54.99 mg/Kg; *p* = 0.000013; summarized in Table S1). In the case of Zn, although women in the exposed group exhibited slightly higher levels than men, this difference was not statistically significant.

Toxic element analysis revealed significantly higher Al concentrations in exposed women compared to exposed men (75.44 ± 39.09 mg/Kg vs. 52.80 ± 30.27 mg/Kg; *p* = 0.000844; Table S2). These findings underline the presence of gender-specific disparities in the bioaccumulation of certain elements among individuals from the exposed population. However, when comparing exposed women to unexposed women, as well as exposed men to unexposed men, no significant differences were observed for Cu, Fe, Zn or Al. These results highlight that gender-specific differences in element concentrations are more pronounced within the exposed group, potentially reflecting distinct exposure pathways or physiological factors that influence the bioaccumulation of these elements.

Next, we aimed to investigate potential correlations between hair elemental concentrations and genotoxicity parameters such as MNBNs, NPBs and NBUDs using a comprehensive approach. Considering that individuals from ASGM areas might exhibit specific clusters of interacting elements, we hypothesized that some essential elements could interact with toxic elements in varying proportions, potentially influencing chromosomal instability and DNA damage. To explore these interactions, we conducted a Spearman correlation analysis, and the resulting correlation matrix is presented in Figure S2.

The analysis revealed correlations between hair elements and genotoxicity parameters (MNBNs, NPBs and NBUDs). For exposed individuals, MNBNs showed significant correlations with Mn, V and Zn, highlighting a potential link between these elements and chromosomal instability.

In the upper and middle sections of the matrix, we assessed associations among toxic and essential elements, aiming to identify shared exposure routes across populations [59]. Distinct patterns emerged between exposed and non-exposed individuals. Of particular interest was a robust and statistically significant cluster involving Sr, V and Fe, with strong positive correlations: Sr-V (*r* = 0.87), V-Fe (*r* = 0.77) and Sr-Fe (*r* = 0.79). These findings suggest that these elements may share common exposure pathways or are influenced by similar environmental or occupational factors, such as soil micromineral deposition [60].

In the exposed group, clusters of Ba with Cu, Mn, Fe, V, Sr, Co and Zn were observed. Additionally, clusters involving Al with Sr and Zn, as well as Ni with Pb, were prominent. The results were a slight indication of ASGM and mining activities [61]. However, the observed multicollinearity and the presence of differential clustering patterns prompted us to extend our analysis using non-linear approaches to better capture the complexity of these interactions.

### 3.4. Principal Component Analysis (PCA) Loadings

We used PCA to resolve collinearity by transforming correlated variables into a smaller set of uncorrelated principal components. This approach has been widely used in environmental studies [62] and allows us to identify the primary sources of variability in the dataset while preserving the integrity of common patterns among the elements [63]. The results of the PCA provided a more accurate representation of the dominant contributors to environmental pollution and served as a basis for linking these components to their potential health effects.

**Figure 3:**
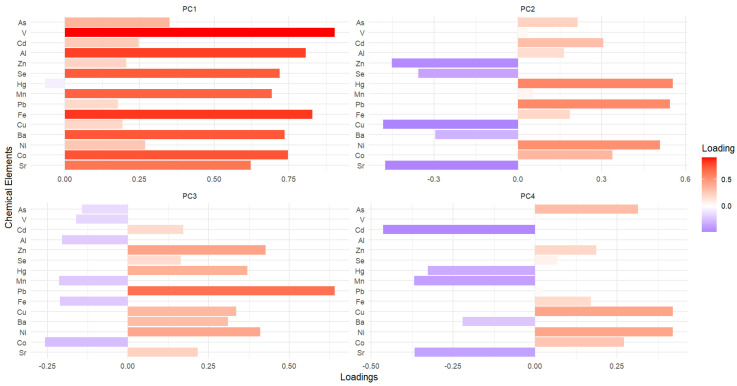
Loadings of chemical elements in the first four principal components. The plots display the contributions (loadings) of each element to the first four principal components (PC1–PC4). Positive loadings (red) indicate elements that are strongly associated with a given component, while negative loadings (blue) reflect opposing contributions.

The principal component (PC) loadings are shown in Figure 3. A total of four components were retained because their cumulative explained variance exceeded the 60% threshold established in the study. This threshold provided a balance between simplifying the data and retaining sufficient variability. The four components explained 65.1% of the variance, with individual contributions of 33.4%, 13.7%, 9.7% and 8.2% for PC1, PC2, PC3 and PC4, respectively. The elbow criterion observed in the cumulative variance curve further supports selecting these components, as the additional components contributed minimally to the total variance (Figure S1).

#### 3.4.1. PC1: Soil-Derived Mining-Associated Element

Principal Component 1 (PC1) had the highest contribution to the total variance, explaining 33.4% of the variability in the dataset. The elements strongly loaded in PC1-V— Fe, Al, Co, Ba, Se and Mn—are well-documented markers of mining-related contamination, particularly in regions affected by ASGM [64]. These elements are commonly mobilized during mining processes and soil disturbance, which are prominent activities in regions with ASGM. Mining operations often result in the erosion of mineral-rich soils and the dispersion of these elements into surrounding ecosystems through runoff and sediment transport [65]. This significant contribution reflects the dominance of elements primarily associated with soil disturbance and mining activities, highlighting their environmental relevance in the residents of the study area.

The ingestion and inhalation pathways play a critical role in the accumulation of metals in human tissues, with water and dietary intake from contaminated crops representing significant exposure routes [66,67]. Among these, V is of particular concern due to its multiple exposure pathways and associated health risks [68]. However, evidence specifically addressing V exposure in ASGM populations remains limited. Our findings suggest that elevated hair V levels in these populations may originate from an anthropogenic or biogenic source yet to be identified. Comparatively, hair V levels in La Mojana population are similar to those observed in residents exposed to petrochemical plants and oil processing activities [69,70]. This similarity may point to the environmental impact of ASGM wastewater, possibly resulting from spills or the deliberate dumping of lubricating oils due to mishandling in ASGM operations [71]. Further investigation through comprehensive environmental monitoring is essential to better understand and address these potential sources of V exposure, including contributions from unidentified biogenic sources.

Similarly, Mn has been identified in elevated concentrations in the hair of ASGM miners, indicating significant exposure through mining activities [72]. Our findings demonstrate a notably high Mn bioavailability, which warrants attention, as the Mn levels observed in this study are more than double those reported for reference populations. These levels are comparable to those found in occupationally exposed ASGM miners and exceed typical Mn exposure levels in the general population [72]. Elevated hair Mn levels were an anticipated finding in the sampled areas of this study. Our previous report encountered elevated Mn concentrations in populations from the El Bagre-Nechí ASGM complex near La Mojana, underscoring the chronic exposure to Mn in these mining-impacted regions [43]. The hair Mn levels reported here raise significant health concerns, particularly for children in La Mojana, given the established association between elevated Mn exposure and reduced cognitive performance [73]. These findings underscore the need for further assessments to evaluate and mitigate the health risks in these vulnerable populations.

The primary sources of Al and Fe contamination in ASGM populations include exposure to neurotoxic metalloids from mining activities, metal scrapping, smelting processes and e-waste recycling [74]. Additionally, consumption of locally grown crops, which may absorb these elements from the soil, contributes to the contamination [66]. Inhalation of dust and direct dermal contact with contaminated materials during mining activities also play a role in the accumulation of these metals in human hair among ASGM miners and associated communities [66].

#### 3.4.2. PC2: Mining-Related Heavy Metals

PC2 explains 13.7% of the total variance and is characterized by strong positive loadings for Hg, Pb and Ni. These elements are widely recognized as toxic metals strongly associated with ASGM and significant implications for environmental contamination and human health [66]. Hg is widely used in gold amalgamation, leading to its release into the environment via volatilization and contamination of soils, water and air [75,76]. Similarly, Pb contamination results from mining tailings, atmospheric deposition and leaching into water sources, which is common in ASGM regions [77]. Considering the Ayapel sampling area and its proximity to the Sinú River basin, the presence of ferronickel mines suggests that Ni contamination may also be influenced by ferronickel extraction and processing, contributing to Ni release through soil disturbance, water contamination and atmospheric emissions [43,78].

In addition to mining activities, these metals have dietary sources that contribute to their accumulation in the local population. Hg is commonly found in fish, especially predatory species, due to its bioaccumulation as methylmercury (MeHg) in aquatic ecosystems, which is of particular concern in fishing-dependent communities [43].

Our results for Hg levels in La Mojana are consistent with previous studies [42], highlighting fish as a major source of Hg exposure among residents. Notably, 34% of the studied population had Hg levels exceeding the permissible limits set by the US Environmental Protection Agency (USEPA); however, while fishing remains a primary pathway for Hg exposure, we cannot rule out other contributing factors. Socioeconomic challenges may drive some fishermen to engage in alternative livelihoods, such as “barequeo” in ASGM areas. We hypothesize that this dual exposure—through subsistence fishing and ASGM activities—substantially increases the local population’s Hg bioaccumulation risk [79].

A notable feature of this PC is the appearance of Se with a loading on the opposite axis to Hg, Pb and Ni, highlighting a distinct behavior that warrants further discussion. The inverse relationship between Se and Hg reflects their well-documented antagonistic interactions [80]. Se is known to form highly stable Hg–Se complexes, reducing the bioavailability and toxicity of Hg. This antagonism plays a crucial role in mitigating Hg-induced damage in biological systems [81]. Se, an essential micronutrient, supports antioxidant defense systems and may provide some protective effects against the oxidative stress and genotoxicity caused by Hg exposure [82]. The separation of Se from Hg, Pb and Ni in PC2 also suggests its predominant origin in diet, agricultural practices or naturally selenium-rich soils, as opposed to mining and industrial activities [83].

#### 3.4.3. PC3: Agricultural and Mining-Related Contaminant Elements

PC3 accounted for 9.7% of the total variance and had strong positive loadings for Pb, Zn and Ni. While active ASGM operations characterize the area, it is also home to subsistence agricultural activities that contribute additional sources of contamination through the use of fertilizers, pesticides and soil disturbance [84]. The previous reported use of phosphate fertilizers, especially superphosphate, introduces metals such as Cu, Ni, Pb, Cd and Zn into regions soils, which can accumulate depending on crop type, fertilizer application rates and flooding or irrigation frequency [85]. In addition, pesticides and fungicides containing Cu, Zn and Hg further contribute to the metal burden in agricultural soils and water [86]. The frequent use of these inputs in local agriculture, coupled with runoff and soil disturbance from mining activities, creates a synergistic pathway for metal contamination in the environment [84]. These practices not only impact the soil but also result in the leaching of metals into water bodies, which are critical for irrigation and local diets. Such contamination elevates the risk of bioaccumulation in crops and other food sources, contributing to dietary exposure pathways. In particular, metals like Pb and Zn, often linked to fertilizers and pesticides, have been reported in agricultural produce grown in contaminated soils, reinforcing the connection between subsistence farming and environmental contamination [87].

#### 3.4.4. PC4: Tailing-Associated Elements

PC4 accounts for 4.2% of the total variance. It is dominated by Cu, Ni and As, elements commonly associated with mining tailings and residual contamination from industrial activities. These metals are often released into the environment during the extraction and processing of minerals, especially in regions affected by ASGM. Tailings, which are often rich in heavy metals, are a significant source of contamination for soils and water systems, especially in areas that rely on these resources for agriculture and domestic use [88]. As, a toxic metalloid, is often leached from tailings into groundwater [89], while Cu and Ni are deposited in soils where they can accumulate and disrupt microbial communities, soil fertility and plant health [90,91]. These metals also pose risks to human health through bioaccumulation in food chains, highlighting the need for effective remediation strategies and sustainable land management to minimize the impact of tailings on ecosystems and local communities [92].

Consistent with our findings, previous studies have reported elevated levels of Cu and Ni in sediments from the Magdalena River, a major contributor to the La Mojana region [93]. Both Cu and Ni concentrations exceeded threshold and probable effect levels, indicating potential ecological risks. Similarly, our analysis identified Cu and Ni as significant contributors to PC3 and PC4, suggesting that the environmental deposition of these metals may be driving increased bioaccumulation in the exposed population.

### 3.5. Main Effects of PCs and DNA Damage and Chromosomal Instability Parameters

Next, we analyzed the relationship between the PCA and the observed frequencies of DNA damage biomarkers in the study population. By correlating specific environmental contaminant profiles with genotoxic outcomes, we aim to elucidate the potential health risks associated with exposure to these pollutants and underscore the importance of environmental monitoring and public health interventions.

We used a non-linear GAMLSS model to evaluate the influence of the different MNBNs, NBUDs and NPsB and the PCs comprising all element loadings. The resulting model is shown in Table 3.

The analysis of MNBN frequencies in the total population revealed significant associations with several factors, highlighting the impact of exposure and demographic variables. Exposure (living in the exposed area) was strongly associated with a 26% increase in MNBN frequencies (PR: 1.26; 95% CI: 1.02–1.57; *p* = 0.039), emphasizing its role as a critical determinant of genotoxic damage. Sex was consistently a risk factor for increased MNBN frequencies (PR: 0.51; 95% CI: 0.41–0.64; *p* < 0.001), probably reflecting the previous results in Table 2. Additionally, specific environmental contaminant profiles (PC2, PC3 and PC4) exhibited significant protective effects, with reductions in MNBN frequencies of 11%, 16% and 17%, respectively.

Building on this general observation, the analysis of the mining and environmental contaminant profiles associated with PCs (PC1, PC2, PC3, PC4) in the exposed population revealed more pronounced associations with genotoxic damage biomarkers, particularly MNBNs.

Demographically, sex continued to show a protective effect in men (PR: 0.53; 95% CI: 0.41–0.69; *p* < 0.001), while age demonstrated a slight but significant increase in MNBN prevalence (PR: 1.01; 95% CI: 1.00–1.02; *p* = 0.034). Among the profiles, PC1 (Soil-Derived Mining-Associated Elements) exhibited the strongest association with MNBNs (PR: 10.45; 95% CI: 9.75–12.18; *p* < 0.001), followed by PC3 (Agricultural and Mining-Linked Elements, PR: 5.85; 95% CI: 5.26–6.50; *p* < 0.001), PC2 (Mining-Related Heavy Metals, PR: 5.32; 95% CI: 4.83–5.85; *p* < 0.001) and PC4 (Tailing-Associated Contaminants, PR: 3.34; 95% CI: 3.01–3.72; *p* < 0.001).

In comparison, the biomarkers NPBs and NBUDs showed more limited associations and lower sensitivity. For NPBs, significant associations were observed only with PC2 (PR: 5.32; 95% CI: 4.83–5.85; *p* < 0.001) and PC3 (PR: 5.85; 95% CI: 5.26–6.50; *p* < 0.001). For NBUDs, the only significant association was with PC2, with a PR of 1.27 (95% CI: 0.95–1.69; *p* < 0.001). These findings confirm the superior sensitivity of MNBNs in detecting genotoxic damage in populations exposed to environmental contaminants, emphasizing its utility as a monitoring tool compared to NPBs and NBUDs.

The marked correlation between MNBNs and PCs represents an interesting finding, in concordance with previous research from our group [43]. In our recent study, we determined that MNBN frequencies were associated with combined exposure to Se, Mn, Hg, Pb and Ni in ASGM areas from the Sinú River basin. Similarly, the findings described here indicate that Se, Mn, Hg, Pb and Ni are mostly clustered in PC1, 2 and 3. Due to the extensive connection through water bodies, we can infer that part of the MNBN frequencies evidenced in residents from La Mojana are due to ASGM activities from the Sinú River basin but might include other contributions or activities that are concomitant with ASGM activities.

To be precise, the intensive ASGM activities might induce extensive soil deposition. When analyzing PC1 in the GAMLSS model, the high MNBN was mostly correlated with V exposure. V compounds have been shown to induce DNA damage and chromosome instability, particularly through the formation of MNBNs, which are indicative of chromosomal aberrations. Studies have demonstrated that V can cause both structural and numerical chromosomal aberrations, with a significant increase in micronucleus formation observed in human lymphocytes and mouse bone marrow cells [94,95]. Additionally, vanadium (IV) has been shown to cause DNA damage via free radical reactions, specifically through the hydroxylation of 2′-deoxyguanosine to form 8-hydroxyl-2′-deoxyguanosine (8-OHdG), a marker of oxidative DNA damage [96]. The cytogenetic effects of V are further supported by in vivo studies showing increased chromosomal aberrations and decreased mitotic indices in cells exposed to vanadium oxides [94,97].

Other PC1 clustered elements such as Co might influence DNA damage. Previous evidence demonstrated Co increased DNA single-strand breaks (DNA-SSB) in human mononuclear blood cells [98], which was also similarly encountered in vivo [99]. However, there is a pressing need to analyze how Co exposure might occur under ASGM population settings and it warrants further research. Similar to Co, another element, Ba, needs more studies. The evidence linking Ba directly to DNA damage is less clear. Some studies have investigated the effects of Ba compounds on gene expression in rat lungs, suggesting potential genotoxic effects, which also was similarly encountered in vivo [99].

In particular, essential elements such as Al and Mn with increased loadings in PC1 are related to DNA damage. Evidence of Al-induced DNA double-strand breaks and chromosomal abnormalities has been seen in mammalian cells [100]. Additionally, Mn has shown increased ROS induction and DNA damage in several models [101]. Even our previous research is in concordance with previous findings.

The analysis of PC2 reveals distinct associations with MNBN frequency across the total, reference control and exposed populations groups, highlighting the differential impacts of this component. In the total population, PC2 shows an inverse association with MNBN frequency (PR = 0.89, 95% CI: 0.81–0.97, *p* = 0.009), suggesting a potential mitigating effect on chromosomal instability. This finding could reflect the protective role of Se, negatively loaded on PC2, known to counteract Hg toxicity by forming stable Hg-Se complexes, reducing oxidative stress, and protecting DNA integrity. However, in the exposed population, PC2 exhibits a strong positive association with MNBN frequency (PR = 5.32, 95% CI: 4.83–5.85, *p* < 0.001), underscoring the genotoxic impact of mining-related heavy metals, including Hg, Pb and Ni. These metals can induce genotoxic effects through various mechanisms, including the inhibition of DNA repair processes [102] and ROS generation [103]. In contrast, no significant association was observed in the unexposed individuals (PR = 0.91, 95% CI: 0.73–1.14, *p* = 0.420), suggesting that the absence of direct environmental exposure limits the influence of PC2 elements on MNBN variability. These findings emphasize the complex interplay between toxic and protective elements in PC2, reflecting the dual impact of environmental and dietary sources.

**Figure 4:**
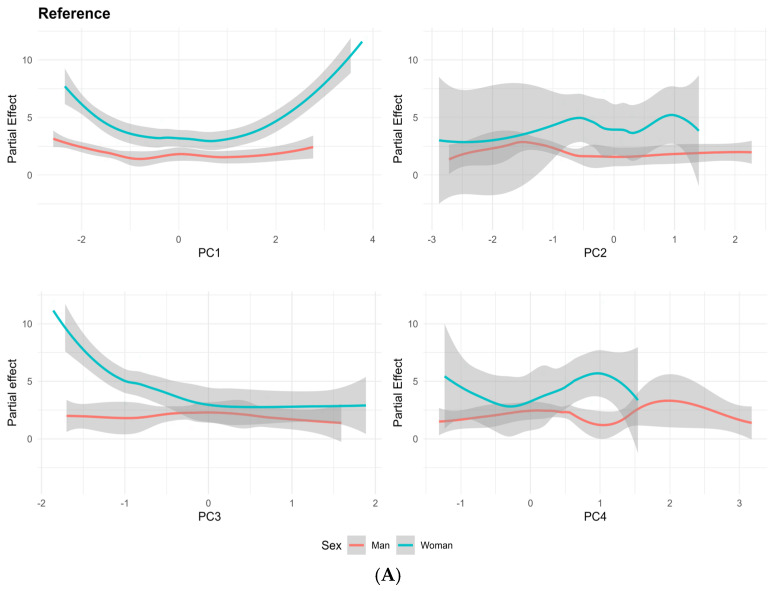

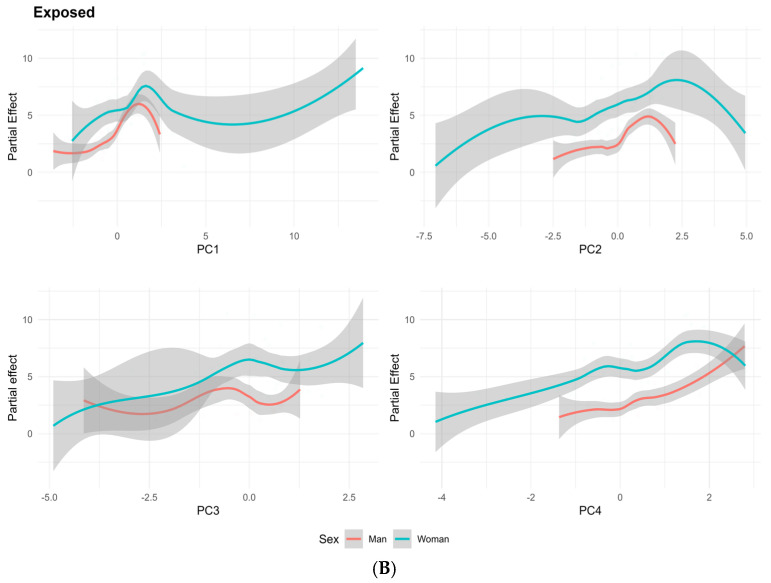
Non-linear effects of principal components on MNBNs, differentiated by sex, according to GAMLSS model. Panel (**A**) represents reference group, while panel (**B**) illustrates exposed population.

In the total population, PC3 shows a statistically significant inverse association with MNBN frequency (PR = 0.84, 95% CI: 0.76–0.93, *p* < 0.001), suggesting a potential protective role at moderate exposure levels. This protective effect may be related to Zn and Cu, essential micronutrients that support antioxidant defenses and DNA repair mechanisms, mitigating oxidative stress and reducing DNA damage [104]. However, in the exposed population, PC3 demonstrates a strong positive association with MNBN frequency (PR = 5.85, 95% CI: 5.26–6.50, *p* < 0.001), underscoring the genotoxic effects of higher exposure to metals such as Pb and Ni, which are prominent in mining and agricultural contamination (Figure 4). At elevated levels, Pb disrupts chromosomal segregation during mitosis, inhibits DNA repair pathways and generates oxidative stress, leading to DNA damage [105] and increased MNBN formation [106]. Similarly, Ni induces DNA–protein crosslinking and impairs repair enzymes [107], further exacerbating chromosomal instability [108].

Excessive levels of Zn and Cu, while typically protective, can contribute to oxidative stress through Fenton-like reactions at high concentrations [109], amplifying the genotoxic effects of Pb and Ni through synergistic interactions (Figure 4). In contrast, in the unexposed group, PC3 shows a more moderate association (PR = 0.74, 95% CI: 0.59–0.91, *p* = 0.009), reflecting the predominance of Zn and Cu at lower exposure levels and minimal contributions from Pb and Ni. This highlights the role of environmental exposure in shaping the impact of PC3 elements on DNA damage markers.

In the exposed population, PC4, associated with mining tailings contaminants, demonstrated a significant association with increased genetic damage biomarkers. Specifically, PC4 was linked to a 17% increase in MNBNs (PR: 1.17; 95% CI: 1.10–1.25; *p* < 0.001), a 12% rise in NPBs (PR: 1.12; 95% CI: 1.05–1.19; *p* = 0.002) and a 15% elevation in NBUDs (PR: 1.15; 95% CI: 1.08–1.23; *p* < 0.001).

Cu, a key element in PC4, plays a dual role at the cellular level, acting as both a pro-oxidant and antioxidant [110]. While it serves as a vital cofactor in metabolic enzymes, excess free copper generates ROS through Fenton-like reactions, exacerbating oxidative stress [111]. This leads to glutathione depletion and significant damage to proteins and DNA [112], contributing to genetic instability and the formation of genotoxic biomarkers. These mechanisms highlight copper’s complex influence on oxidative damage and defense [113].

Arsenic (As) is another significant contributor to PC4 and further amplifies the genotoxic risks associated with mining tailings. Epidemiological studies have linked prolonged exposure to inorganic arsenic (iAs) with severe health outcomes, including cancers of the skin, bladder and lungs, as well as cardiovascular diseases and atherosclerosis [114]. Additionally, previous studies have associated genetic instability in human cells with elevated levels of As and Hg, elements commonly found in mining-impacted areas [115]. At the cellular level, As disrupts redox homeostasis by inhibiting critical antioxidant enzymes, such as glutathione peroxidase (GPx) and glutathione reductase (GR). This inhibition depletes intracellular glutathione levels, resulting in an accumulation of ROS. The ensuing oxidative stress leaves cells highly vulnerable to DNA damage, further exacerbating genetic instability and increasing the risk of mutation-driven pathologies [116].

### 3.6. Exposure-Dependent and Sex-Specific Trends Across PC

The partial effect analysis, visualized in Figure 4, illustrates the exposure-dependent and sex-specific trends in MNBN frequency across the PCs. Adjusted for age and stratified by sex, the data highlight a consistent pattern: women exhibit higher MNBN frequencies compared to men across all PCs, irrespective of their exposure status (reference or exposed groups). This trend is evident in the observed curves, where the MNBN frequency remains elevated for women at lower PC loadings, suggesting that DNA damage in women may be triggered at lower elemental concentrations or under conditions of minimal bioaccumulation. The PC1 cluster appears to have a pronounced effect on women, indicating a possible association between its elements and increased MNBN frequencies at low doses. Although studies addressing gender-related exposure in ASGM contexts are limited, there is evidence suggesting that certain elements within PC1, such as Fe and V, can induce DNA damage even at low bioaccumulation levels [117,118]. Additionally, other components like PC2, PC3 and PC4 appear to elevate MNBN risk among exposed women. Current evidence of low-dose risks is primarily limited to specific elements, such as As [119]. Other PCs such as PC2, PC3 and PC4 seem to increase the MNBN risk among exposed women. Evidence of particular risk at low doses is reduced to some elements, such as As [119].

These findings suggest inherent biological differences, potentially related to hormonal influences or variations in DNA repair mechanisms, that render women more susceptible to genotoxic effects associated with environmental exposures [120]. The lower capacity for base and DNA damage repair in women is a widely documented fact [58,121]. This reduced capacity is directly influenced by hormonal processes that act as key modulators of DNA repair mechanisms [122]. These biological differences represent a critical factor that increases the vulnerability of women exposed to contaminants derived from ASGM systems, intensifying the risks associated with this type of exposure [123].

Additionally, differences in metal metabolism and bioaccumulation, including higher storage of metals like Hg in adipose tissue, may contribute to this disparity. Hg, particularly in its methylated form, accumulates in lipid-rich tissues such as fat. MeHg is a highly toxic, lipophilic organic form of Hg that readily crosses biological barriers, including the blood–brain and placental barriers [124], facilitating its accumulation in lipid-rich tissues such as adipose tissue. This lipophilicity enables MeHg to integrate into fat stores, potentially leading to higher body burdens in individuals with greater adipose reserves [125].

Sex-based differences in adipose tissue distribution and function have been well documented. Women typically possess a higher percentage of body fat compared to men, with fat predominantly distributed subcutaneously in the gluteofemoral region, whereas men tend to accumulate visceral fat in the abdominal area. These differences are influenced by sex hormones, particularly estrogens, which promote subcutaneous fat deposition in women [126].

The higher proportion of adipose tissue in women could predispose them to greater bioaccumulation of lipophilic substances like MeHg. However, the current scientific literature does not provide conclusive evidence that women bioaccumulate more MeHg than men solely based on adipose tissue differences. Factors such as dietary habits, exposure levels and physiological differences in Hg metabolism also play significant roles in MeHg bioaccumulation.

Other metals studied, including Pb, Se and Cd, may also localize in fatty tissues through interactions with lipids or proteins [127,128]. These patterns underscore unique toxic potential in women.

Additionally, social and environmental factors further compound women’s exposure. A study of women in La Mojana identified fish consumption as a primary pathway for Hg exposure, as locally sourced fish contained elevated levels of methylmercury [45]. Drinking water sources were also significant contributors to Hg exposure, reflecting the broader environmental contamination in the region [45].

Women’s roles in cooking and household activities often expose them indirectly to metals through contaminated water and air or tools used in artisanal gold mining [129]. Furthermore, subsistence farming and local diets may contribute additional exposure to Pb and Zn through crops irrigated with contaminated water or grown in polluted soils [130].

Finally, the traditional roles of women in these communities often mean limited access to protective measures or education about exposure risks, exacerbating their vulnerability. Combined with physiological factors, such as lower body weight and a higher proportion of adipose tissue, women may bioaccumulate certain metals more readily. These findings emphasize the need for gender-specific interventions addressing both occupational exposures in men and significant indirect exposures in women. Strategies should include educational campaigns for safer dietary habits, regular environmental monitoring and policy measures to mitigate contamination in water sources and local food chains. These efforts are crucial for reducing the burden of genetic damage and promoting public health in mining-affected regions.

The findings highlight the need for a comprehensive and gender-sensitive approach in public health policies to address exposure risks in communities located in artisanal and small-scale gold mining areas. While previous analyses have prioritized impacts on men due to their involvement in mining and fishing activities, this study, along with [45], reveals that women experience significant levels of exposure, sometimes higher than men. Moreover, the detection of multi-element contamination with toxic substances such as Al, Pb, As, Ni, Cd and Ba underscores the urgency to expand existing programs and policies under Law 1658 of 2013, including the National Unified Mercury Plan (PUNHg) and the Sectoral Environmental Action Plan for Mercury (PASAHg), which currently focus primarily on Hg. These findings support the need to develop a zoning of high-risk ecosystems to prioritize effective public health interventions and preventive measures.

### 3.7. Strengths and Limitations

This study has several strengths that enhance its contribution to the understanding of genotoxic risks in ASGM-affected populations. First, the use of the CBMN-Cyt assay provides a robust and validated approach to assess DNA damage and chromosomal instability, providing reliable biomarkers such as MNBNs, NPBs, and NBUDs. Second, the combined application of PCA and GAMLSS modeling allowed us to explore complex multi-element exposures and their non-linear relationships with genotoxic outcomes. This provided a sensitive understanding of the interplay between essential and toxic metals. Third, the study’s focus on a vulnerable population in La Mojana, an area heavily impacted by ASGM, highlights critical public health issues in a region with limited previous research. However, several limitations associated with our results must be acknowledged. One primary limitation is the small sample size of the population included in the study, which is largely influenced by the inherent conditions of the exposure area. In the case of La Mojana, both geographical features and public security challenges significantly hinder access to exposed populations. Geographically, La Mojana is characterized by flood-prone areas, where high water flow during the rainy season restricts access to certain communities. Furthermore, intensive subsistence fishing practices in the region partially reduce participation, especially in the male population, as men frequently travel across multiple water bodies to improve their chances of catching fish [42]. Public security concerns further exacerbate these challenges; the presence of armed groups in the region has fueled population displacement, limiting the involvement of residents in studies of this nature over recent years. Future studies should prioritize expanding sample sizes through improved logistical strategies.

Another limitation of this study lies in the need to reconcile the population’s metal detoxification characteristics with the bioaccumulation dynamics of the elements analyzed using GAMLSSs. In a previous study conducted on the population of La Mojana, we found a correlation between MNBN frequency and the presence of the GSTM1null polymorphism, which was associated with Hg levels in the population [131]. However, other elements might also be associated with GSTM1null if they share similar detoxification mechanisms to Hg. For instance, prior evidence has shown that individuals with the GSTM1null genotype have higher levels of As in their blood in various mining exposure contexts [132,133]. Given the multi-element exposure framework of the present study, future research should consider applying GAMLSSs that incorporate the presence of the GSTM1null polymorphism and the concentration dynamics of elements in hair. Additionally, variability in dietary and lifestyle factors, beyond fish consumption, may contribute to differences in metal exposure and detoxification dynamics. Variables such as the consumption of vegetables, fruits, meat and processed foods, as well as water quality and preparation methods, could act as potential confounders. These factors were not explicitly measured in the present study, representing a limitation. Future research should incorporate a more comprehensive dietary assessment to better understand the role of these variables in shaping the associations observed.

## 4. Conclusions

This study highlights the significant genetic damage associated with multi-elemental exposure in populations affected by ASGM in the La Mojana region of Colombia. Using the DNA damage and chromosome instability parameters of the CBMN-Cyt assay and PCA, we identified key environmental and dietary pathways contributing to DNA damage and chromosomal instability. The findings demonstrate a pronounced amplification of genotoxic effects in exposed populations, with women consistently showing higher MNBN frequencies compared to men. This gender disparity suggests distinct exposure pathways, such as dietary habits involving contaminated fish and crops, and the potential for differential accumulation of toxic metals like Hg, Pb and Ni.

The interplay of essential and toxic metals, as reflected in the PCA components, underscores the dual role of these elements, protective at moderate levels but highly genotoxic at elevated concentrations. Elevated levels of mining-related contaminants, including V, Fe and Al, were strongly associated with increased MNBN frequencies, emphasizing the compounded impact of environmental contamination on health.

These findings call for urgent public health interventions tailored to mining-affected regions, with a focus on gender-specific strategies to mitigate exposure. Efforts should include improved monitoring of environmental and dietary contaminants, remediation of contaminated areas and education on reducing exposure risks. Addressing these challenges is critical for safeguarding vulnerable populations from the long-term health impacts of ASGM-related contamination.

The statistical approach employed was essential for analyzing the non-linear and combined effects of chemical element concentrations on biomarkers. Integrating methods such as PCA and GAMLSSs allows us to address the complexity of interactions among chemical elements and overcome issues like multicollinearity, ensuring a more precise and robust analysis.

## Figures and Tables

**Table 1:** Main demographic characteristics of studied population: individuals from exposed and reference areas.

Group	Sampling Areas		Total
	Reference	Exposed	
**Number of individuals**	37	71	
**Individuals by area (Department)**			
Cotorra-Reference area (Córdoba)	37	-	37
Magangué (Bolivar)	-	15	15
Ayapel (Córdoba)	-	16	16
Caimito (Sucre)	-	40	40
**Gender N (%)**			
Women	20 (54.05)	39 (54.92)	59 (54.62)
Men	17 (45.94)	32 (45.07)	49 (45.37)
**Age (mean ± S.D)**	36.51 ± 9.71	35.49 ± 11.25	35.84 ± 10.71
**Occupation N (%)**			
**Women**			
Housewives	20 (54.05)	39 (54.92)	59 (54.62)
**Men**			
Fishermen	-	32 (45.07)	32 (45.09)
Peasants	17 (45.94)	-	17 (15.74)
**Consumption habits**			
**Alcohol consumption N (%)**			
Non-alcohol consumers	34 (91.89)	62 (87.32)	96 (94.74)
Alcohol consumers *			
Low	3 (8.10)	7 (9.85)	10 (9.25)
Moderate	-	2 (2.81)	2 (1.85)
**Tobacco consumption N (%)**			
Smokers (%)	2 (5.40)	6 (8.45)	8 (7.40)
Non-tobacco smokers (%)	35 (94.59)	65 (91.54)	100 (92.59)
**Fish intake days/week N (%)**			
1–2 (Low)	8 (20.51)	11 (15.49)	19 (17.59)
3–4 (Medium)	16 (41.02)	30 (42.25)	46 (42.59)
5–7 (High)	13 (38.46)	30 (42.25)	43 (39.81)
**Vegetable consumption N (%)**			
Green vegetables	32 (86.48)	56 (78.87)	98 (81.48)
Fruits	5 (13.51)	15 (21.12)	20 (18.51)

* According to NIAAA (National Institute of Alcohol Abuse and Alcoholism); S.D: Standard deviation.

**Table 2:** Non-parametric comparison by Mann–Whitney U test of CBMN-Cyt assay parameters by gender between exposed and reference areas.

Parameters	Reference Area			Exposed Areas			
	N	Mean ± SD	Median	N	Mean ± SD ^a^	Median	*p*-Value
			(25th–75th)			(25th–75th)	
**MNBN**							
Women	20	5.19 ± 5.04 ^a^	3.0 (2.5–6.5)	39	6.59 ± 4.63 ^a^	6.0 (3.0–8.0)	NS
Men	17	2.05 ± 2.63	1.0 (0.0–3.0)	32	4.28 ± 2.56	3.0 (3.0–5.0)	≤0.01
Total	37	2.52 ± 2.12	3.0 (1.0–3.2)	71	**5.54 ± 3.98**	**3.0 (3.0–8.0)**	**≤0.01**
**NBUDs**							
Women	20	0.80 ± 1.60	0.0 (0.0–1.0)	39	0.46 ± 1.02	0.0 (0.0–0.0)	NS
Men	17	0.35 ± 0.78	0.0 (0.0–0.5)	32	0.68 ± 1.28	0.0 (0.0–1.0)	NS
Total	37	0.29 ± 0.62	0.0 (0.0–1.0)	71	0.56 ± 1.14	0.0 (0.0–1.0)	NS
**NPB**							
Women	20	0.58 ± 0.94	0.0 (0.0–1.0)	39	2.6 ± 5.45 ^a^	1.0 (0.0–2.7)	≤0.01
Men	17	0.56 ± 0.80	0.0 (0.0–1.0)	32	0.58 ± 1.73	0.0 (0.0–0.0)	NS
Total	37	0.54 ± 0.87	0.0 (0.0–1.0)	71	**1.59 ± 3.96**	**1.0 (0.0–1.0)**	**≤0.05**

**Bold** for statistically significant difference compared to individuals from reference areas. ^a^ *p* ≤ 0.01 when different from men with the same exposure status; NS: Not statistically significant.

**Table 3:** Prevalence ratios (PR) with 95% confidence intervals for MNBNs, NBUDs and NPBs across total population, reference and exposed groups adjusted for PC, exposure, sex and age using generalized additive models for location, scale and shape (GAMLSSs) with Poisson distribution for response.

	Total				Reference				Exposed			
		CI95%				CI95%				CI95%		
MNBN	PR	Lower Limit	Higher Limit	*p*-Value	PR	Lower Limit	Higher Limit	*p*-Value	PR	Lower Limit	Higher Limit	*p*-Value
Exposure	**1.26**	**1.02**	**1.57**	**0.039**								
Sex	**0.51**	**0.41**	**0.64**	**<0.001**	**0.58**	**0.36**	**0.96**	**0.042**	**0.53**	**0.41**	**0.69**	**<0.001**
Age	1.01	1.00	1.02	0.108	0.97	0.95	1.00	0.074	**1.01**	**1.00**	**1.02**	**0.034**
PC1	1.03	0.99	1.07	0.135	1.1	0.96	1.25	0.180	**10.45**	**9.75**	**12.18**	**<0.001**
PC2	**0.89**	**0.81**	**0.97**	**0.009**	0.91	0.73	1.14	0.420	**5.32**	**4.83**	**5.85**	**<0.001**
PC3	**0.84**	**0.76**	**0.93**	**<0.001**	**0.74**	**0.59**	**0.91**	**0.009**	**5.85**	**5.26**	**6.5**	**<0.001**
PC4	**0.83**	**0.75**	**0.92**	**<0.001**	0.92	0.72	1.18	0.521	**3.34**	**3.01**	**3.72**	**<0.001**
**NBUDs**												
Exposure	1.23	0.67	2.24	0.499								
Sex	1.00	0.56	1.80	0.988	0.65	0.16	2.56	0.539	1.24	0.61	2.50	0.551
Age	**1.04**	**1.01**	**1.07**	**0.005**	1.01	0.94	1.09	0.734	**1.05**	**1.02**	**1.08**	**0.004**
PC1	0.99	0.87	1.14	0.912	1.23	0.87	1.73	0.255	1.01	0.86	1.19	0.920
PC2	1.18	0.93	1.49	0.183	0.92	0.5	1.67	0.784	1.27	0.95	1.69	0.111
PC3	0.76	0.58	1.00	0.052	0.63	0.34	1.15	0.145	0.79	0.57	1.08	0.149
PC4	0.85	0.63	1.14	0.270	0.86	0.43	1.7	0.662	0.81	0.52	1.25	0.341
**NPB**												
Exposure	**0.36**	**0.23**	**0.55**	**<0.001**								
Sex	0.53	0.32	0.87	0.014	1.00	0.26	3.8	0.999	1.13	0.55	2.32	0.737
Age	1.01	0.99	1.04	0.356	1.03	0.96	1.1	0.408	1.01	0.98	1.05	0.426
PC1	1.09	0.98	1.2	0.112	**1.55**	**1.14**	**2.09**	**0.009**	1.04	0.85	1.27	0.735
PC2	1.1	0.93	1.3	0.286	**1.97**	**1.16**	**3.35**	**0.019**	1.26	0.94	1.69	0.125
PC3	0.92	0.75	1.12	0.398	1.16	0.8	1.67	0.436	0.71	0.48	1.05	0.092
PC4	1.07	0.89	1.3	0.465	**2.88**	**1.69**	**4.89**	**<0.001**	**0.62**	**0.45**	**0.86**	**0.005**

**Bold** for statistically significant effect.

## Data Availability

Data are unavailable to protect the sensitive information of certain ethnic communities located in gold mining areas.

## Supplementary Materials

The following supporting information can be downloaded at: https://www.mdpi.com/article/10.3390/toxics13030202/s1, Figure S1: Explained and cumulative variance of the principal components. The bar plot (left) shows the percentage of variance explained by each principal component (PC), while the line plot (right) illustrates the cumulative variance. The dashed line represents the 60% cumulative variance threshold established in the study, exceeded by the first four components, accounting for 65.1% of the total variance; Figure S2: Spearman correlation matrix for residents, both unexposed (reference) and exposed, living near ASGM operations in northern Colombia. The primary parameters analyzed include chromosomal instability (MNBN), DNA damage (NPBs and NBUDs) and concentrations of essential and toxic elements; Table S1: Essential element concentrations in mg/Kg from hair samples obtained from unexposed controls and exposed residents located in proximity to ASGM operations in North Colombia. Data are expressed as mean ± SD (standard deviation), median (25th and 75th percentile) and both minimum and maximum values encountered. Bold for statistically significant difference compared to individuals from reference areas; “a” *p* ≤ 0.05; “b” *p* ≤ 0.01; “c” *p* ≤ 0.001 when different from men with the same exposure status; Table S2: Toxic elements concentration in mg/Kg from hair samples obtained from unexposed controls and exposed residents located in proximity to ASGM operations in north Colombia. Data are expressed as mean ± SD (standard deviation), median (25th and 75th percentile) and both minimum and maximum values encountered. Bold for statistically significant difference compared to individuals from reference areas; “a” *p* ≤ 0.05; “b” *p* ≤ 0.01 when different from men with the same exposure status.
