# Supplementary material for: Genetic Damage and Multi-Elemental Exposure in Populations in Proximity to Artisanal and Small-Scale Gold (ASGM) Mining Areas in North Colombia
Source: Toxics. 2025 Mar 11;13(3):202. doi: 10.3390/toxics13030202 (PMC11946375; doi:10.3390/toxics13030202)
Supplement: Supplementary file 1 [file toxics-13-00202-s001.zip › toxics-3412919-supplementary.pdf]

Figure S1. Explained and cumulative variance of the principal components. The bar plot (left) shows the percentage of variance explained by each principal component (PC), while the line plot (right) illustrates the cumulative variance. The dashed line represents the 60% cumulative variance threshold established in the study, exceeded by the first four components, accounting for 65.1% of the total variance.

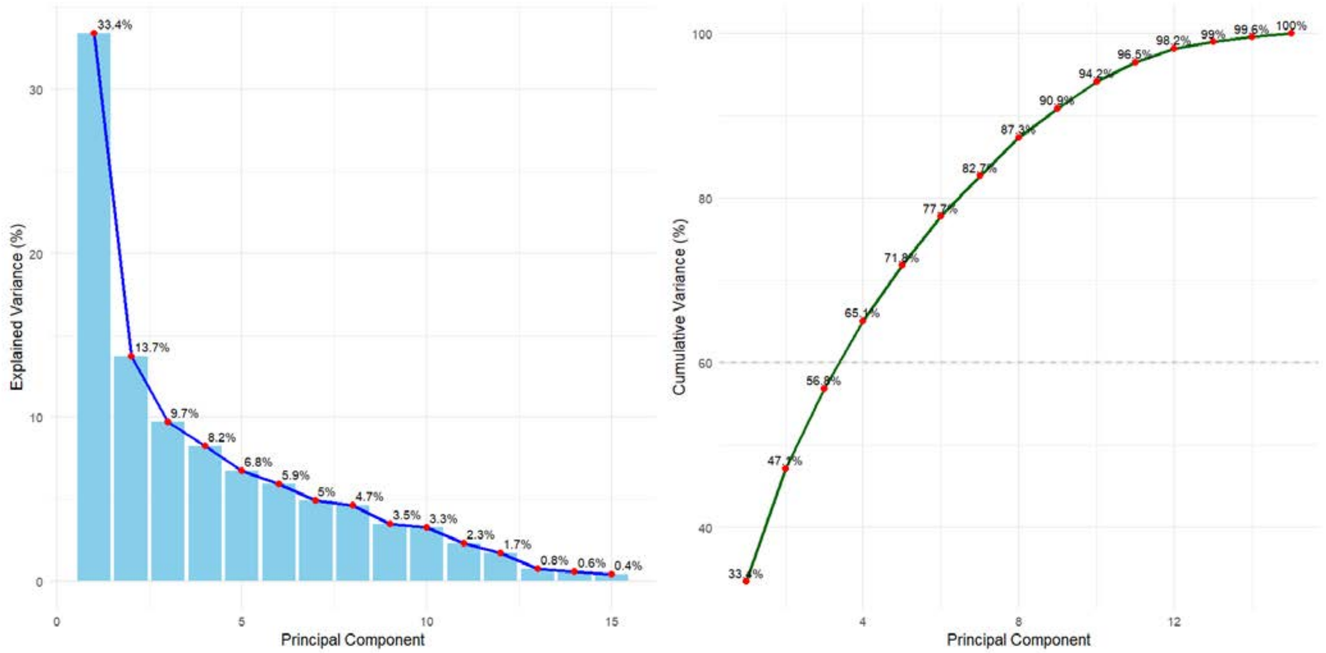

Figure S2: Spearman correlation matrix for residents, both Unexposed (Reference) and Exposed, living near ASGM operations in northern Colombia. The primary parameters analyzed include chromosomal instability (MNBN), DNA damage (NPB and NBUDs), and concentrations of essential and toxic elements. Red squares indicate positive correlations, while blue squares denote negative correlations. The numbers within the squares represent Spearman correlation coefficients. Significant correlations are marked with an asterisk (*), and a double asterisk (**) signifies a p-value ≤ 0.01.

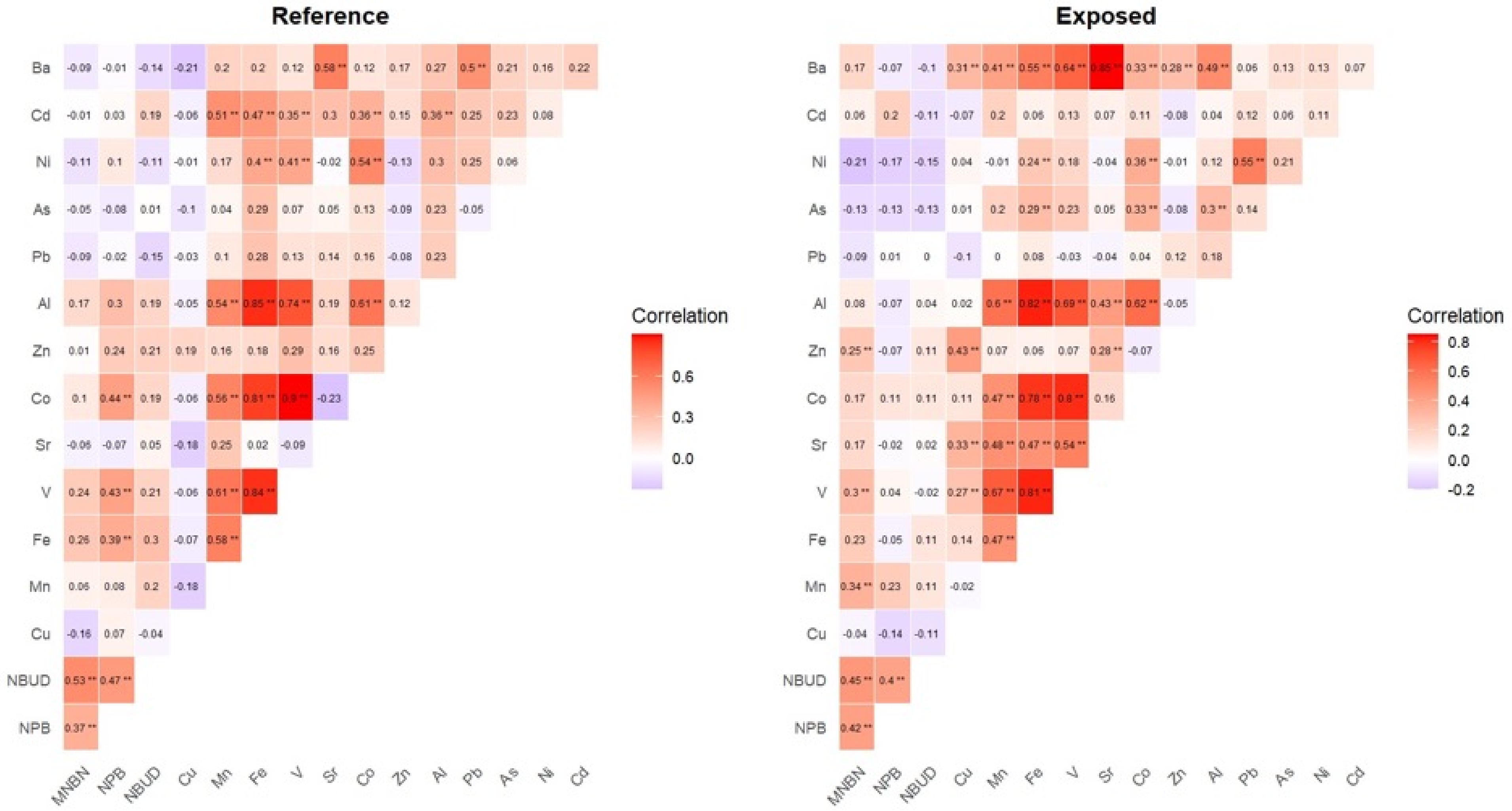

Table S1: Essential elements concentration in mg/Kg from hair samples obtained from unexposed controls and exposed residents located in proximity to ASGM operations in north Colombia. Data are expressed as mean ± SD (standard deviation), median (25th and 75th percentile) and both minimum and maximum values encountered. **Bold** for statistically significant difference compared to individuals from reference areas; "a" P≤ 0,05; "b" P≤ 0,01; "c" P≤ 0,001 when different from men with the same exposure status.

Elements	Unexposed controls				Exposed residents				p-value
	N	Mean ± SD	Median (25th – 75th)	Min – Max	N	Mean ± SD	Median (25th – 75th)	Min – Max	
**Cu**									
Women	20	23.55± 25.93	16.01 (11.01 – 19.49)	8.26 – 98.30	39	**51.08 ± 109.40**[a]	15.02 (10.12 – 21.65)	6.31 – 540.90	0.006
Men	17	45.61± 127.8	15.02(8.88 – 21.91)	6.31 – 540.9	32	22.23 ± 51.43	9.19 (6.33 – 14.92)	6.25 – 2.86	0,005
Total population	37	33.68 ± 87.98	15.58 (9.65 – 20.54)	6.31 – 540.9	71	38.08 ± 88.76	11.20 (8.26 – 20.62)	6.25 – 540.9	0.800
**Mn**									
Women	20	7.73 ± 4.71	7.33 (3.86 – 9.49)	1.59 – 18.29	39	12.26 ± 8.51	10.13 (6.77 – 16.58)	1.59 – 47.11	0,654
Men	17	7.14± 4.59	6.22(2.97– 10.47)	1.59– 16.21	32	9.87 ± 6.14	8.39 (1.15 – 12.41)	3.11 – 31.47	0,746
Total population	37	7.46 ± 4.60	7.33 (3.36 – 10.05)	1.59 – 18.29	71	11.19 ± 7.58	9.09 (6.77 – 14.67)	1.59 – 47.11	**0.007**
**Fe**									
Women	20	109.8± 51.15	105.3 (67.89 – 137.03)	50.14 – 265.6	39	**109.20 ± 53.53**[a]	94.90 (75.73 – 122.90)	47.99 – 324.50	0,954
Men	17	80.02 ± 54.99	67.05(44.31– 98.26)	33,35 – 265.06	32	74.79 ± 26.46	67.05 (62.07 – 88.15)	21.75 – 138.80	0,534
Total population	37	96.10 ± 54.32	87.13 (61.57 – 119.6)	33.35– 265.6	71	93.68 ± 46.51	83.63 (65.27 – 115.80)	21.75 – 324.50	0.800
**V**									
Women	20	0.33 ± 0.15	0.28 (0.23– 0.42)	0.11 – 0.77	39	0.47 ± 0.27	0.41 (0.31 – 0.54)	0.12 – 1.52	0,989
Men	17	0.24 ± 0.12	0.22 (0.13 – 0.32)	0.10– 0.51	32	0.30 ± 0.11	0.29 (0.23 – 0.40)	0.11 – 0.48	0,994
Total population	37	0.29 ± 0.14	0.27 (0.17 – 0.37)	0.10 –0.77	71	0.39 ± 0.23	0.39 (0.27 – 0.47)	0.11 –1.52	**0.011**
**Sr**									
Women	20	7.29 ± 3.51	6.80 (4.93– 10.18)	2.07 – 15.37	39	8.71 ± 5.30	7.34 (5.41 – 10.98)	2.07 – 28.30	0,888
Men	17	6.86± 2.37	6.90 (5.28 – 8.78)	2.98 – 10.98	32	8.31 ± 4.33	6.99 (5.04 – 11.30)	2.07 – 16.51	0,863
Total population	37	7.10 ± 3.00	6.90 (5.13 – 8.78)	2.07 – 15.36	71	8.53 ± 4.86	7.05 (5.13 – 10.98)	2.07 – 28.30	0.100
**Co**									
Women	20	0.08 ± 0.05	0.06 (0.05 – 0.10)	0.02 – 0.2	39	0.10 ± 0.06	0.09 (0.04 – 0.11)	0.03 –0.33	0,998
Men	17	0.06 ± 0.04	0.04 (0.03 – 0.08)	0.02 – 0.18	32	0.06 ± 0.03	0.06 (0.03 – 0.09)	0.01 – 0.14	0,999
Total population	37	0,07 ± 0.05	0.05 (0.03 – 0.10)	0.02 – 0.22	71	0.08 ± 0.05	0.06 (0.04 – 0.10)	0.01 – 0.33	0.444
**Se**									
Women	20	1.22 ± 0.46	1.15 (0.95 – 1.29)	0.85 – 3.03	39	1.30 ± 0.70	1.16 (1.00 – 1.31)	0.70 – 4.23	0,993
Men	17	1.17 ± 0.38	1.06 (0.93 – 1.33)	0.63 – 1.88	32	0.98 ± 0.20	0.99 (0.85 – 0.11)	0.63 – 1.56	0,981
Total population	37	1.22 ±0.42	1.13 (0.94 – 1.27)	0.63 – 3.03	71	1.15 ± 1.06	0.92 (1.23 – 0.63)	0.63– 4.23	0.690
**Zn**									
Women	20	164.2 ± 91.19	138.2 (94.94 – 219.8)	62.22 – 420.6	39	147.10 ± 63.13	127.50 (101.9 – 151.0)	93.36 – 420.60	0,090
Men	17	145.5 ± 36.29	136.7 (124.5 – 167.00)	96.19 – 218.8	32	138.0 ± 52.86	124.60 (101.90 – 142.90)	88.09 – 337.70	0,374
Total population	37	155.6 ± 71.16	137.6 (115.5 – 191.4)	62.22 – 420.6	71	143.00 ± 58.50	124.60 (101.90 – 146.50)	88.09 – 420.60	0.325

Table S2: Toxic elements concentration in mg/Kg from hair samples obtained from unexposed controls and exposed residents located in proximity to ASGM operations in north Colombia. Data are expressed as mean ± SD (standard deviation), median (25th and 75th percentile) and both minimum and maximum values encountered. **Bold** for statistically significant difference compared to individuals from reference areas; "a" P≤ 0,05; "b" P≤ 0,01 when different from men with the same exposure status.

Elements	Unexposed controls				Exposed residents				p-value
	N	Mean ± SD	Median (25th – 75th)	Min – Max	N	Mean ± SD	Median (25th – 75th)	Min – Max	
**Al**									
Women	20	75.44 ± 39.09	70.45 (47.47 – 97.57)	20.26 – 163.50	39	74.04± 70.58	65.19 (31.31 – 84.37)	11.90 – 439.20	0,890
Men	17	52.80 ± 30.27	39.74 (28.56 – 65.57)	18.48 – 122.10	32	47.75 ± 22.59	39.74 (29.09 – 63.92)	11.90 – 88.07	0,549
Total population	37	65.04 ± 36.63	61.80 (34.34 – 86.06)	18.48 – 163.50	71	62.19 ± 55.71	58.72 (31.31 – 78.41)	11.90 – 439.20	0.779
**Pb**									
Women	20	3.67 ± 3.50	2.50 (1.62 – 3.93)	0.36 – 12.27	39	4.23 ± 4.44	3.41 (1.73 – 5.83)	0.36 – 25.98	0,955
Men	17	3.27 ± 4.02	1.48 (0.60 – 5.62)	0.36 – 14.25	32	4.75 ± 4.26	3.64 (1.74 – 5.40)	0.36 – 16.02	0,860
Total population	37	3.49 ± 3.70	2.30 (0.77 – 3.90)	0.36 – 14.25	71	4.47 ± 4.33	3.64 (1.73 – 5.83)	0.36 – 25.98	0.245
**Hg**									
Women	20	0.55 ± 0.57	0.26 (0.19 – 0.94)	0,09 – 1.81	39	0.87 ± 0.86	0.49 (0.23 – 1.60)	0.06 – 3.25	0,974
Men	17	0.50 ± 0.70	0.21(0.16– 0.55)	0.06 – 3.02	32	1.32 ± 1.03	1.42 (0.35 – 1.81)	0.06 – 3.25	0,922
Total population	37	0.53 ± 0.62	0,23 (0.17 – 0.55)	0.06 – 3.02	71	1.07 ± 0.96	0.51 (0.26 – 1.77)	0.006 – 3.25	**0.002**
**As**									
Women	20	0.23 ± 0.18	0.16 (0.11 – 0.26)	0.09 – 0.76	39	0.34 ± 0.46	0.18 (0.11 – 0.30)	0.006 – 2.74	0,991
Men	17	0.24 ± 0.23	0.16 (0.10 – 0.25)	0.07 – 0.83	32	0.17 ± 0.11	0.16 (0.11 – 0.21)	0.06 – 0.76	0,993
Total population	37	0.23 ± 0.20	0.16 (0.11 – 0.25)	0.07– 0.83	71	0.26 ± 0.35	0.17 (0.11 – 2.24)	0,06 – 2.74	0.654
**Ni**									
Women	20	0.91 ± 0.78	0.64 (0.47 – 1.04)	0.33 – 3.84	39	1.42 ± 2.87	0.77 (0.46 – 1.05)	0.28 – 18.03	0,959
Men	17	0.78 ± 0.46	0.73 (0.40 – 0.93)	0.33 – 1.90	32	1.06 ± 1.40	0.56 (0.38 – 0.98)	0.23 – 6.45	0,973
Total population	37	0.85 ± 0.65	0.68 (0.47– 1.04)	0.33 – 3.84	71	1.26 ± 2.32	0.61 (0.43 – 1.05)	0.23 – 18.03	0.298
**Cd**									
Women	20	0.21 ± 0.23	0.28 (0.11 – 0.31)	0,01 – 0.43	39	0.23 ± 0.23	0.19 (0.08 – 0.32)	0.01 – 1.10	0,997
Men	17	0.12 ± 0.10	0.09 (0.02 – 0.19)	0.01 – 0.32	32	0.15 ± 0.12	0.11 (0.04 – 0.27)	0.02 – 0.43	0,88
Total population	37	0.16 ± 0.12	0.13 (0.03 – 0.27)	0.01 – 0.43	71	0.20 ± 0.19	0.16 (0.04 – 0.27)	0.01 – 1.10	0.382
**Ba**									
Women	20	7.66 ± 4.38	6.29 (4.76 – 9.01)	2.30 – 20.08	39	11.75 ± 8.99	9.29 (7.69 – 12.93)	1.49 – 53.51	0,687
Men	17	7.80 ± 3.67	6.85 (4.92 – 9.06)	3.09 – 15.32	32	9.03 ± 5.71	7.93 (4.74 – 13.26)	1.49 – 21.37	0,996
Total population	37	7.72 ± 4.01	6.85 (4.83 – 9.06)	2.30 – 20.08	71	10.52 ± 7.76	8.83 (5.44 – 12.93)	1.40 – 53.51	**0.042**
